# Apical Ischemia Is a Universal Feature of Apical Hypertrophic Cardiomyopathy

**DOI:** 10.1161/CIRCIMAGING.122.014907

**Published:** 2023-03-21

**Authors:** Rebecca K. Hughes, João B. Augusto, Kristopher Knott, Rhodri Davies, Hunain Shiwani, Andreas Seraphim, James W. Malcolmson, Shafik Khoury, George Joy, Saidi Mohiddin, Luis R. Lopes, William J. McKenna, Peter Kellman, Hui Xue, Maite Tome, Sanjay Sharma, Gabriella Captur, James C. Moon

**Affiliations:** 1 Institute of Cardiovascular Science (R.K.H., J.B.A., K.K., R.D., H.S., A.S., G.J., L.R.L., W.J.M., G.C., J.C.M.), University College London, United Kingdom.; 2 MRC Unit for Lifelong Health and Ageing (R.D., G.C.), University College London, United Kingdom.; 3 Barts Heart Centre, The Cardiovascular Magnetic Resonance Imaging Unit and The Inherited Cardiovascular Diseases Unit, St Bartholomew’s Hospital, West Smithfield, London, United Kingdom (R.K.H., J.B.A., K.K., R.D., H.S., A.S., J.W.M., G.J., S.M., L.R.L., J.C.M.).; 4 Cardiology Department, Hospital Professor Doutor Fernando Fonseca, Amadora, Portugal (J.B.A.).; 5 William Harvey Institute, Queen Mary University of London, United Kingdom (J.W.M., S.M., M.T., S.S.).; 6 Cardiovascular Clinical and Academic Group, Molecular and Clinical Sciences Institute, St. George’s University of London, United Kingdom (S.K.); 7 Instituto de Investigación Biomédica de A Coruña, Spain (W.J.M.).; 8 National Heart, Lung, and Blood Institute, National Institutes of Health, Department of Human and Health Services, Bethesda, MD (P.K., H.X.).; 9 Department of Cardiology, Inherited Heart Muscle Conditions Clinic, Royal Free Hospital, NHS Trust, United Kingdom (G.C.).

**Keywords:** apical hypertrophic cardiomyopathy, cardiomyopathy, humans, hypertrophic, hypertrophy, stroke volume

## Abstract

Apical hypertrophic cardiomyopathy (ApHCM) accounts for ≈10% of hypertrophic cardiomyopathy cases and is characterized by apical hypertrophy, apical cavity obliteration, and tall ECG R waves with ischemic-looking deep T-wave inversion. These may be present even with <15 mm apical hypertrophy (relative ApHCM). Microvascular dysfunction is well described in hypertrophic cardiomyopathy. We hypothesized that apical perfusion defects would be common in ApHCM.

**Methods:**

A 2-center study using cardiovascular magnetic resonance short- and long-axis quantitative adenosine vasodilator stress perfusion mapping. One hundred patients with ApHCM (68 overt hypertrophy [≥15 mm] and 32 relative ApHCM) were compared with 50 patients with asymmetrical septal hypertrophy hypertrophic cardiomyopathy and 40 healthy volunteer controls. Perfusion was assessed visually and quantitatively as myocardial blood flow and myocardial perfusion reserve.

**Results:**

Apical perfusion defects were present in all overt ApHCM patients (100%), all relative ApHCM patients (100%), 36% of asymmetrical septal hypertrophy hypertrophic cardiomyopathy, and 0% of healthy volunteers (*P*<0.001). In 10% of patients with ApHCM, perfusion defects were sufficiently apical that conventional short-axis views missed them. In 29%, stress myocardial blood flow fell below rest values. Stress myocardial blood flow was most impaired subendocardially, with greater hypertrophy or scar, and with apical aneurysms. Impaired apical myocardial blood flow was most strongly predicted by thicker apical segments (β-coefficient, −0.031 mL/g per min [CI, −0.06 to −0.01]; *P*=0.013), higher ejection fraction (−0.025 mL/g per min [CI, −0.04 to −0.01]; *P*<0.005), and ECG maximum R-wave height (−0.023 mL/g per min [CI, −0.04 to −0.01]; *P*<0.005).

**Conclusions:**

Apical perfusion defects are universally present in ApHCM at all stages. Its ubiquitous presence along with characteristic ECG suggests ischemia may play a disease-defining role in ApHCM.

**Clinical Perspective**

Hypertrophic cardiomyopathy is defined by unexplained hypertrophy. Of the various morphologies, apical hypertrophic cardiomyopathy appears characteristic with differences in the genetics, ECG, and outcomes. This article demonstrates that all patients with apical hypertrophic cardiomyopathy, with both early and advanced disease, have apical ischemia. This ubiquitous finding highlights its potential future use as an imaging biomarker that may help the diagnosis in mild disease and may also be a candidate therapeutic target. Its presence cannot be a risk factor on its own (being ubiquitous), but there is scope to further understand how the extent or depth of ischemia links to disease progression (scar and aneurysms) and risk.

Hypertrophic cardiomyopathy (HCM) affects 1 in 500 people and has genotypic and phenotypic heterogeneity. Defined by unexplained left ventricular hypertrophy (LVH) ≥15 mm,^1,2^ the apical variant (apical HCM [ApHCM]) is diagnosed when LVH predominates in the left ventricular (LV) apex. ApHCM is the most distinctive of the HCM morphologies with ethnic differences, an increased prevalence in athletes, a lower prevalence of sarcomeric gene mutations, characteristic structural features including apical scar and aneurysm formation, and an altered risk profile with more atrial fibrillation/stroke but similar mortality.^3–7^

One characteristic feature is the distinctive ECG, with precordial T-wave inversion, sometimes giant, in almost all patients.^4,8^ While these partly mirror large QRS complexes,^9^ the ECG is sometimes misinterpreted as acute coronary syndrome because it looks ischemic. But the role of ischemia in ApHCM remains ill defined.

Cardiovascular magnetic resonance (CMR) has advantages in diagnosing ApHCM because it visualizes the apex and apical pathology, such as scar and apical aneurysms, better than echocardiography.^10–12^ This can be used to demonstrate extended ApHCM phenotypic features including its milder variant relative ApHCM, where there is loss of usual apical myocardial tapering, systolic apical cavity obliteration, and wall thickness for apical segments that is unequivocally increased, but fails to reach 15 mm.^4,13,14^ Microvascular dysfunction is a known feature of HCM and is thought to be multifactorial with contributions from reduced capillary density, vascular remodeling, impaired autoregulation, interstitial fibrosis, disarray, and extravascular compression due to ventricular hypertrophy and diastolic dysfunction.^15^ HCM perfusion defects are seen by nuclear scintigraphy and computed tomography positron emission tomography,^16,17^ more recently also by CMR perfusion mapping, which permits stress and rest quantification.^18,19^ Using this, early disease has been explored, with perfusion defects demonstrated before overt hypertrophy in gene carriers, suggesting ischemia is an early feature of phenotype development.^20^ In ApHCM, impaired apical perfusion is well described but not systematically studied.^16,17,21^ Given the above insights, we hypothesized that apical microvascular ischemia demonstrated by perfusion mapping (including long-axis mapping for distal defects) might be common in both early and advanced ApHCM and have important associations.

## Methods

A prospective study approved by the National Health Service Research Ethics Committee and Health Research Authority and conducted in accordance with the Declaration of Helsinki. All subjects provided written, informed consent (REC 18/LO/0188 and 17/SC/0077). The data that support the findings of this study are available from the corresponding author upon reasonable request.

### Study Population

A total of 199 study patients were prospectively recruited into this study. N=109 patients with ApHCM or relative ApHCM were recruited from tertiary referral cardiomyopathy clinics and directly through screening referrals for CMR at St Bartholomew’s Hospital and St George’s University Hospital, London, United Kingdom. The apical variant of HCM was defined as maximum apical wall thickness ≥15 mm in the end diastole, with apical wall thickness exceeding basal; subclassified as pure, with isolated apical hypertrophy; and mixed, with both apical and septal hypertrophy, but with apex the thickest.^4^ Relative ApHCM was defined previously^13^ as inappropriate apical hypertrophy compared with expected apical wall thickness (loss of apical tapering, apical thickness >basal thickness) but <15 mm and other characteristic features of the disease (distinctive ECG changes,^9^ apical cavity obliteration,^21^ or apical aneurysm). Other inclusion criteria were as follows: (1) age ≥18 years, (2) no secondary causes of LVH, and (3) no known coronary artery disease. Of these, 9 patients were later excluded due to failure of acquisition of perfusion maps due to loss of ECG gating (n=3), lack of stress map reconstruction (n=2), substandard image quality due to significant arrhythmia (n=1), inadequate stress (n=1), concomitant hypertension/overlap disease (n=1), and concomitant ischemic heart disease, unknown prior to recruitment (n=1). Thus, the final cohort of patients with overt ApHCM (n=68) and relative ApHCM (n=32) with available stress perfusion CMR data was n=100. In addition, there were 2 comparator groups: (1) n=40 healthy volunteers (HVs) with no significant medical history, including cardiovascular disease and (2) n=50 known nonapical HCM (with asymmetrical septal hypertrophy [ASH], recruited in the same way as ApHCM). Exclusion criteria were the presence of conventional contraindications to CMR. Patients with permanent pacemakers/internal cardiac defibrillators were excluded from this study.

### CMR Acquisition

CMR scans were performed at the Barts Heart Centre and Chenies Mews Imaging Centre on a 1.5T magnet (Aera; Siemens Healthcare, Erlangen, Germany) using a standard clinical protocol modified for ApHCM deep phenotyping. The protocol consisted of cine imaging (balanced steady-state free precession), native T1 mapping (using a modified look-locker inversion recovery sequence, 5 s [3 s] 5 s with motion correction), T2 mapping (motion correction single-shot balanced steady-state free precession), stress and rest perfusion, late gadolinium enhancement (LGE) with motion correction and phase-sensitive inversion recovery, and postcontrast T1 mapping. Synthetic extracellular volume fraction was derived from the native and postcontrast T1 maps. T1, T2, and extracellular volume mapping was performed for basal, mid, and apical short-axis and 2 long-axis slices (2 and 4 chamber).

Adenosine vasodilator stress perfusion was performed using a standard clinical approach (adenosine [140 μg/kg per min, increased to 175 μg/kg per min for a further 2 minutes if <10 bpm heart rate increase or no symptoms]). A gadolinium-based contrast agent (gadoterate meglumine, Dotarem; Guerbet, Paris, France) was injected into a peripheral vein during peak vasodilator stress at 0.05 mmol/kg. Sixty images were typically acquired for basal, mid, and apical LV short-axis slices and a 2-chamber long-axis slice. When the heart rate exceeded 100 bpm, the 4 slice groups could not be imaged within a single concatenation and therefore taken over 2 concatenations instead.^22^ Rest perfusion images were subsequently acquired after 6 to 10 minutes. Perfusion mapping was implemented using the Gadgetron streaming software image reconstruction framework.^23^ In 8 cases, the long-axis view was not performed (and was misplanned in one other case), but the short-axis views demonstrated perfusion defects in all cases, so the scans were not repeated.

### CMR Analysis

CMRs were analyzed using commercially available software (CVI42; Circle Cardiovascular Imaging, Calgary, Canada). For parametric analysis of T1, T2, and extracellular volume maps, LV endocardial and epicardial contours were manually drawn using the 3 short-axis slices. Borders were offset by 10%, and models for both global and segmental (16-segment American Heart Association model) were created for each parameter. Visual perfusion defects were assessed from both conventional and mapping images (including the 2-chamber long-axis slice). For parametric map analysis of stress and rest myocardial blood flow (MBF), LV endocardial and epicardial contours were applied using machine learning with human oversight using a 16-segment model. Endocardial and epicardial subsegmentation was also automated by sequentially offsetting each border by 50%. In scans where the blood pool was not visible due to cavity obliteration, contours were manually drawn as the model was not trained for this.^24^

LV volume analyses were performed using a validated machine learning algorithm.^25^ LV maximum wall thickness (MWT) was also measured using a validated machine learning algorithm in end diastole using the short-axis cine stack.^26^ LGE was quantified using the full-width half-maximum technique with LGE expressed in grams and as a percentage of total myocardium and apical LGE reported as relative enhanced area (%). An apical aneurysm (>5 mm) or microaneurysm (<5 mm) was defined by the presence of an akinetic/dyskinetic motion, scarring, and a nonobliterating apical cavity typically distal to an area of obliteration. Systolic apical cavity obliteration was measured using previously defined criteria.^13^

### Electrocardiograms

Standard 12-lead ECG was performed with assessment of T-wave negativity and R-wave amplitude.

### Statistical Analysis

Statistical analysis was performed in SPSS (IBM SPSS statistic, version 26.0). Normality of data was assessed on histograms and using the Shaprio-Wilk test. Normally distributed and non-normally distributed continuous data were presented as mean±SD or median and interquartile range, respectively, and compared across participant groups, using independent Student *t* test or Mann-Whitney-Wilcoxon test. Categorical data were presented as counts and percentage and compared using the χ^2^ test. Correlation was assessed with Pearson coefficient if normally distributed or Spearman correlation if non-normally distributed. Linear regression was used to determine which factors were associated with apical stress MBF in patients with overt and relative ApHCM. All clinical parameters were proposed for inclusion in the univariate models. Unique, clinically relevant predictor variables with a *P* value <0.10 on univariate analysis were then entered into a final multivariable regression base model that adjusted for age, sex, and body surface area (BSA), using a forward stepwise procedure. Final models had a variance inflation factor <3 indicating no significant multicollinearity between model variables. A 2-sided *P* value ≤0.05 was considered significant.

## Results

**Figure 1.**
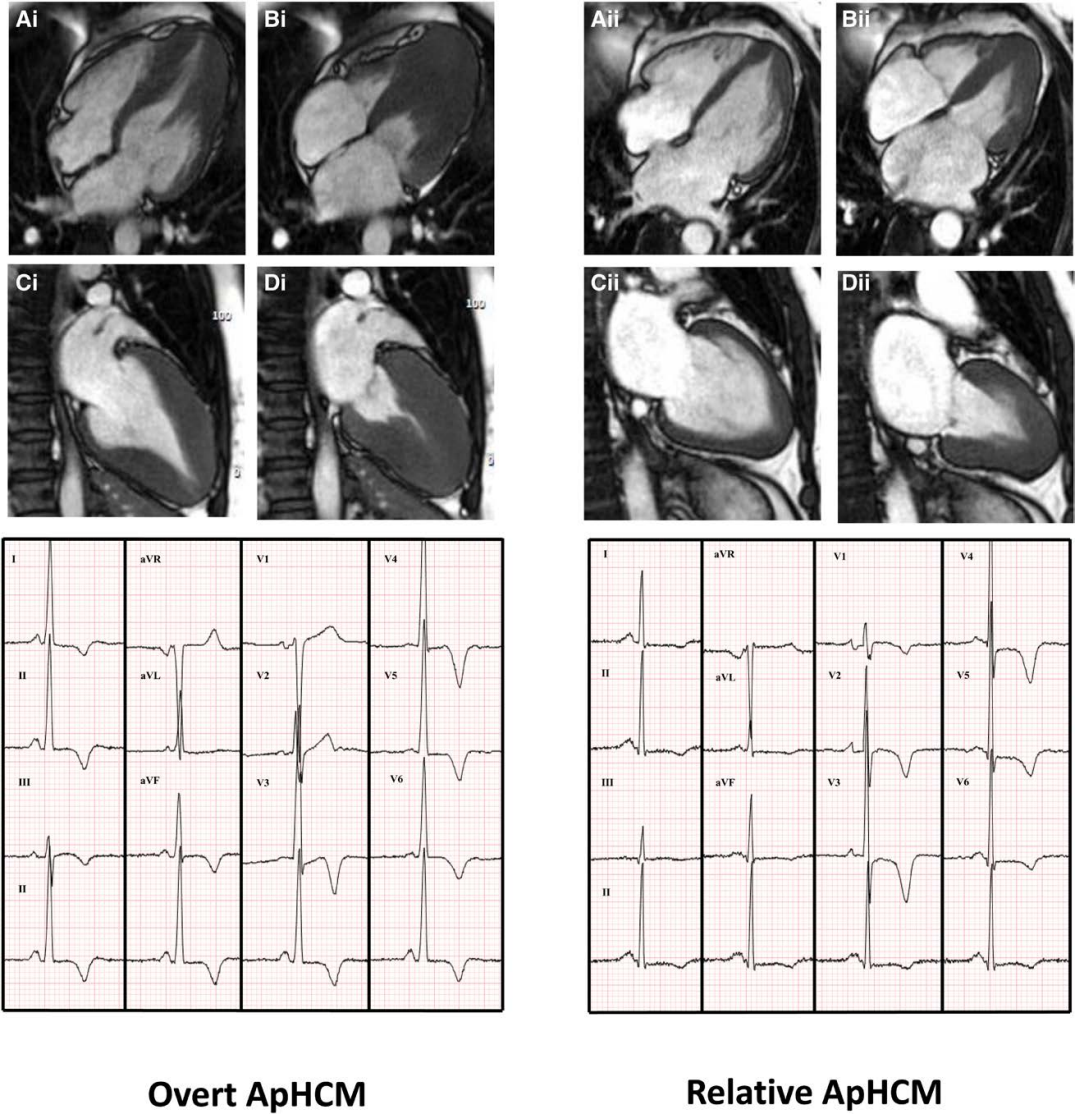
**Long-axis cardiac magnetic resonance and ECG appearances of overt versus relative apical hypertrophic cardiomyopathy (ApHCM).** Images depict overt ApHCM on the left (**Ai-Di**) and relative ApHCM on the right (**Aii-Dii**). End-diastolic frames of 4-chamber (**Ai, Aii**) and 2-chamber (**Ci, Cii**) show apical hypertrophy and "ace of spades" appearance of left ventricular cavity (more marked in overt disease). End-systole (**Bi, Bii, Di, Dii**) demonstrate apical cavity systolic obliteration, extending more basally in overt disease. ECGs show similar appearances with deep T-wave inversion in the precordial leads.

One hundred patients with ApHCM underwent quantitative perfusion CMR (Table 1; Table S1). Of these, 68 met wall thickness criteria for ApHCM (MWT, ≥15 mm; age, 58.6±13; 74% men; BSA, 2.02±0.3 m^2^) and were subclassified as pure ApHCM (n=39; 57%) or mixed ApHCM (n=29; 43%). Thirty-two subjects met criteria for relative ApHCM (similar morphological changes but apical MWT <15 mm^13^; age, 55.4±14; 84.4% men; BSA, 1.97±0.2 m^2^; Figure 1). They were compared with 50 ASH HCM subjects (age, 51.8±15; 58% men; BSA, 2.01±0.2 m^2^) and 40 HVs (age, 42.9±15; 58% men; 1.94±0.2 m^2^). Thirty-six of 50 ASH HCM patients had genetic testing performed, of whom 25 (69%) had a causal mutation identified. Thirty-five of 100 ApHCM patients had genetic testing performed. As expected, a lower number had causal genetic mutations, here 8 (22%). Comorbidities were prevalent in all patient groups (diabetes, hypertension, and hypercholesterolemia) but not (by definition) in HVs. The only significant between-group difference was more diagnosed hypercholesterolemia in overt ApHCM. Investigation for occlusive coronary artery disease had been undertaken clinically in some patients (57% overt, 38% relative, 48% ASH, and 0% HV), which (by our inclusion criteria) was negative in all cases. ECG analyses and medication information are in the Supplemental Material.

**Table 1.**
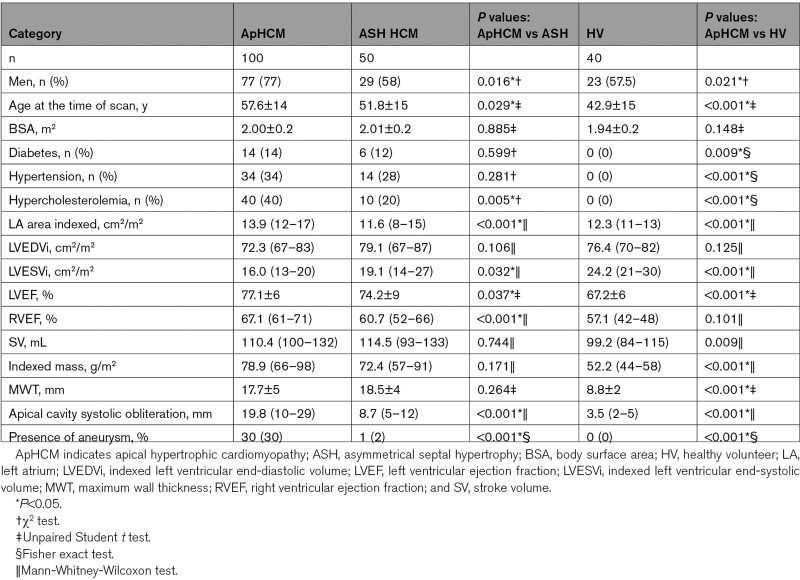
Demographic and Baseline Cardiovascular Magnetic Resonance Characteristics of ApHCM, ASH HCM, and HVs

### Baseline CMR Characteristics

Overt ApHCM had 20.1±4 mm apical hypertrophy, 25.8 (0–32) mm apical cavity obliteration, and an aneurysm in 40%. Relative ApHCM had 12.5±2 mm apical hypertrophy, 18.6 (14–23) mm apical cavity obliteration, and an aneurysm in 9% (all microaneurysms). Left atrial dilatation was common in overt (63%) and relative (59%) ApHCM. ASH HCM had 18.5±4 mm MWT with 38% left atrial dilatation, and only 1 patient had an apical aneurysm (a patient with mid cavity obstruction; Table 1; Table S1).

In overt ApHCM, relative ApHCM, ASH, and HV, respectively, LGE was present in 90%, 41%, 42%, and 0%. Global LGE was quantitatively greater in overt ApHCM compared with both relative ApHCM and ASH (*P*<0.001). T1 was globally slightly higher in overt ApHCM, relative ApHCM, and ASH HCM (1057 [1025–1073], 1010 [991–1036], and 1024 [1002–1049] ms, respectively), compared with HVs (1000 [982–1020] ms; *P*<0.001 for all). ApHCM subjects with apical aneurysms/microaneurysms had a higher apical MWT than those without (19.6±4 versus 16.9±5 mm; *P*=0.012). All aneurysms had LGE (100% versus 65.2%; *P*<0.001).

### Quantitative Perfusion Analysis

**Figure 2.**
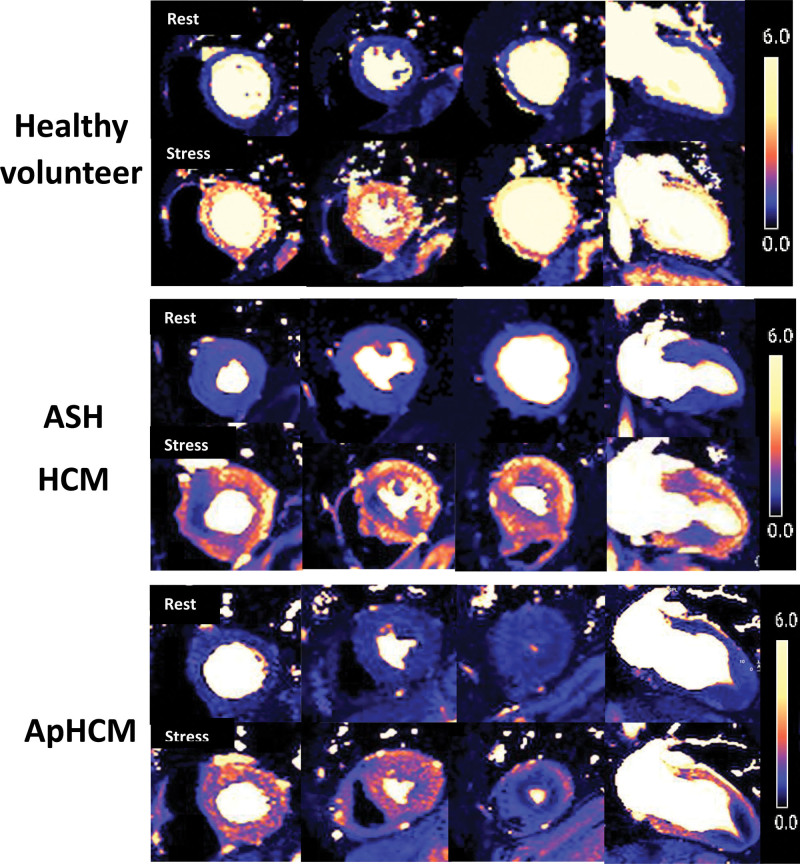
**Stress and rest quantitative perfusion maps in health and disease.** Rest and stress perfusion maps in 3 short-axis and 1 long-axis view. The color scale (**right**) denotes myocardial blood flow in mL/g per min. Rest flow is normal in all groups. During stress, healthy volunteers achieve global hyperemia with no perfusion defects. In asymmetrical septal hypertrophy hypertrophic cardiomyopathy (ASH HCM), while there is hyperemia, there are often dense perfusion defects, mainly in the hypertrophied areas. In apical hypertrophic cardiomyopathy (ApHCM), perfusion defects are seen circumferentially in the apical subendocardium, here with stress flow below rest flow.

**Figure 3.**
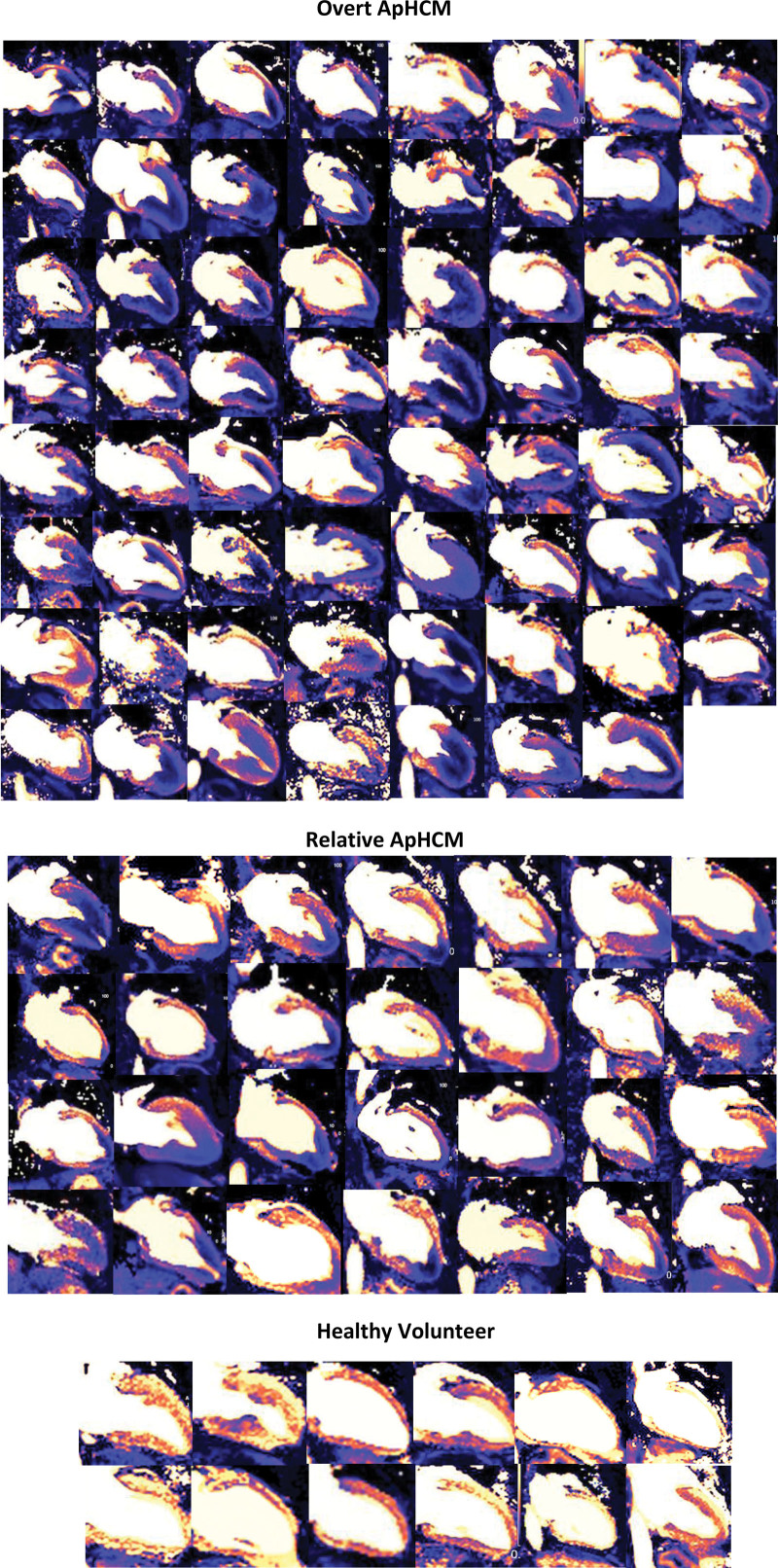
**Two-chamber long-axis stress perfusion maps in all cases of overt and relative apical hypertrophic cardiomyopathy (ApHCM).** The extent of perfusion defects varies, as does the presence of below rest adenosine flow. There were no perfusion defects in healthy volunteer controls.

**Figure 4.**
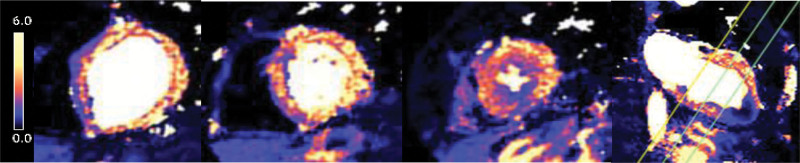
**The importance of apical coverage.** The conventional 3 short-axis approaches here would have missed the apical perfusion seen on the long-axis view, as found in 10% of our cohort.

A typical perfusion data set at rest (top) and stress (bottom) is shown in HVs, ASH, and ApHCM (Figure 2). One hundred percent (68/68) of overt ApHCM and 100% (32/32) of relative ApHCM patients had apical perfusion defects visually. No HV had perfusion defects. Figure 3 shows all long-axis examples for both overt and relative ApHCM with 12 HVs for comparison. Every ApHCM case demonstrates obvious apical hypoperfusion despite vasodilatation in basal segments. The perfusion defects varied in anatomical extent: some were confined just to the apex, extending to mid segments in 38%. In 10%, conventional short-axis perfusion imaging alone missed the perfusion defect captured by the long-axis view, as it was so apical (Figure 4). Eighteen of 50 (36%) ASH HCM subjects also had apical perfusion defects, but in 12 of 18, there was clear apical hypertrophy (mixed septal and apical disease) with subtle hypertrophy in an additional three. Ten of 18 also had classic precordial T-wave inversion.

Globally, stress MBF and myocardial perfusion reserve were lower in all 3 HCM subgroups than HVs, with overt ApHCM and ASH HCM demonstrating the lowest values, particularly in the subendocardium (Table S2). Global resting MBF was lower in all 3 HCM subgroups compared with HVs. The main quantitative reduction in stress MBF and myocardial perfusion reserve in ApHCM was in the apex. The reduction in apical stress MBF was greater in overt than relative ApHCM (1.20 [0.9–1.5] versus 1.57 [1.4–2.0] mL/g per min; *P*<0.001). Both were lower than ASH HCM (1.89 [1.5–2.4] mL/g per min; *P*<0.001 overt; *P*=0.032 relative). The apical:basal gradient (comparison of the lowest stress MBF in any of the 4 apical segments compared with the lowest stress MBF in any of the 6 basal segments) similarly demonstrated a significant difference in regional flow in both overt and relative ApHCM versus ASH HCM (0.66 [0.5–0.8] and 0.75 [0.6–0.9] versus 1.08 [0.9–1.4]; *P*<0.001 for both). Flow was the lowest in the subendocardium, and strikingly, stress apical subendocardial MBF was lower than rest in 25 of 68 (36.7%) overt ApHCM, 3 of 32 (9.4%) relative ApHCM, and 4 of 50 (8%) ASH HCM (Table S2).

### Comparison of Morphological Subtypes

The 30 of 100 ApHCM patients with aneurysms/microaneurysms had lower apical stress MBF than those without (1.20 [1.0–1.3] versus 1.42 [1.1–1.7] mL/g per min; *P*=0.047). There were no differences between mixed or pure ApHCM in either global stress MBF (1.62 [1.4–1.8] versus 1.65 [1.4–1.9] mL/g per min; *P*=0.611) or apical stress MBF (1.23 [1.0–1.5] versus 1.15 [0.9–1.5] mL/g per min; *P*=0.446; Table S3).

### Apical Scar and Therapy

LGE was present in 88% of overt ApHCM and 38% of relative ApHCM. When present, it always involved apical segments, although there could be scar elsewhere (more basal, right ventricular insertion points) in 48% of overt and 3% of relative ApHCM. The distribution of apical LGE is detailed in Figure S1 and Table S4; no subject had ischemic coronary territory LGE suggestive of previous myocardial infarction. Apical stress MBF was lower with more apical scar (R=−0.287; *P*=0.006; scar present versus absent, 1.23 [0.98–1.54] versus 1.51 [1.27–1.98] mL/g per min; *P*<0.001).

β-Blockers were taken in 43 of 100 (43%) ApHCM and 16 of 50 (32%) ASH HCM subjects at the time of scanning. This did not alter HR rise with adenosine (*P*=0.611). Apical stress MBF was lower in β-blocked ApHCM patients (1.22 [0.99–1.47] versus 1.40 [1.14–1.77] mL/g per min; *P*=0.016) but MWT and proportion of apical aneurysms was equivalent.

### Predictors of Apical Stress Flow

Apical stress MBF by univariate analysis (Table S5) was lower with greater MWT, higher indexed LV mass, higher LV and right ventricular ejection fractions, a smaller LV end-systolic volume (indexed), more fibrosis (diffuse: higher global T1, higher global T2, more apical LGE), greater T-wave negativity, and taller R waves on ECG. Multivariable analysis after adjustment for age, sex, and BSA (R^2^ for the model, 0.564; *P*≤0.001) showed age, MWT, LV ejection fraction, and maximum R-wave amplitude on ECG as independent predictors of apical stress MBF (Table S6).

## Discussion

In this, the largest CMR study of ApHCM to date, fully quantitative myocardial perfusion mapping with long-axis imaging was performed to investigate apical perfusion. We anticipated that apical perfusion defects would be common but not the key finding, that apical perfusion defects were ubiquitous in 100% of patients with ApHCM, in both morphologically mild (relatively ApHCM, <15 mm hypertrophy) and advanced disease (scar and apical aneurysm).

When conducting observational studies, identifying 100% prevalence of a phenomenon is an extraordinary finding. In HCM, none of the following commonly measured/observed features are 100% prevalent—sarcomere gene mutations, ECG abnormalities, disarray, or small vessel disease.^27–29^ While macroscopic LVH seems universal, this is definition dependent and can, by strict criteria, be absent (eg, here in relative ApHCM). So, we question the results. First, we wonder whether this is truly 100% prevalent. Statistically, the 95% CI is 3% (rule of 3), so at least 97% of patients are expected to have perfusion defects even if larger studies are conducted. Second, could there be false positives? Possibly, but there were no positives in the HVs, and ASH HCM subjects with apical hypoperfusion never had it in isolation. Third, could there be recruitment bias or overinterpretation? Possibly, but we show the perfusion maps of every ApHCM subject with long-axis images (Figure 3) for reader review, representing zero degrees of freedom for study investigators selection. Some had no long-axis imaging, but the apical short-axis images in these cases demonstrated the perfusion defects.

So, what is the explanation for the 100% (>97%) prevalence in ApHCM? We believe that the observed phenomenon must be tightly linked to the underlying pathophysiology of apical hypertrophy—either causally, as an immediate close consequence of it, or (if there are multiple underlying causes) that there is a final common pathway, which is why it is present at all observable stages of the disease, in young and old male and female patients. As the study was limited to ApHCM, ASH HCM, and HVs, other causes of apical hypertrophy have not been explored, such as Fabry (which was here excluded), but stress perfusion defects in Fabry are not apically limited and stress flow not reported as lower than rest,^30^ something that occurred in 28% of ApHCM overall.

We hypothesize, based on ApHCM ECG changes and other HCM findings, that the perfusion defects are likely to represent microvascular ischemia—a collective term for a process with multiple pathogeneses. The hyperdynamic ApHCM ventricle with early systolic stroke volume ejection,^31,32^ apical cavity obliteration, contractile persistence, and impaired diastolic relaxation^21^ may be drivers, these later processes being potential mechanisms of stress flow being lower than rest. We postulate that the net effect of early and complete apical systolic contraction causes a compressive effect on the microvasculature, and coupled with the impaired diastolic relaxation reducing normal coronary flow, this results in apical ischemia. By whichever mechanism, ischemia may not occur subsequent to LVH, but rather secondary to altered myocardial mechanics, and may drive LVH, accelerating phenotype development. The precise mechanisms require more exploration and experimentation—myocardial mechanics, exercise physiology, alterations during pacing, coronary sinus sampling, longitudinal sampling, advanced ECG techniques could all be studied.

A key clinical task in HCM management is risk stratification. Myocardial ischemia has previously been demonstrated in symptomatic and asymptomatic HCM patients, and its presence postulated to contribute to atrial and ventricular arrhythmias, heart failure, and death, with adverse outcomes potentially following the detection of microvascular dysfunction by years.^33^ The severity of microvascular dysfunction is reportedly relevant to the development of heart failure and with recurrent ischemia and myocyte death resulting in replacement fibrosis, the extent of which relates to the degree of LV impairment,^15^ further mechanistic insights are revealed about the possible natural progression of microvascular ischemia and risk. Here, the presence of apical ischemia alone cannot be differentially prognostic as it is ubiquitous. Yet, quantification, such as the degree of ischemia (extent, proportion, and transmural depth), may be prognostic or may lead to phenotype development (LVH acceleration, scar, or aneurysm formation) and possibly to risk. Here, we note lower apical stress MBF with scar and aneurysms, now a weak indication for implantable cardiac defibrillator insertion.^1,34^ A recent study of 12 overt ApHCM patients using computed tomography positron emission tomography found apical perfusion defects in 83% of subjects and apical scar in 25%.^35^ The 2 patients without apparent ischemia were not described. The high spatial resolution and multiparametric nature of CMR (cines, LGE, and quantitative perfusion) may have advantages, particularly when ischemia can be limited to only the distal apex, and microaneurysms or extensive hypertrophy could confound, if not independently characterized. Understanding the triad of apical hypertrophy, ECG changes and apical ischemia could lead to effective therapies, targeting either microvascular disease or contraction (myosin-binding inhibitors), but this is currently unknown. Lastly, MBF in HCM has not yet been explored with other stressors (eg, exercise). Further work is needed to understand this fully and to appreciate if the degree of ischemia links with disease progression and whether HCM-targeted therapies that reduce hypercontractility and regress LV mass may also alter the extent of apical ischemia and what this signifies in the wider context of the disease.

Study limitations include that this was a single time point imaging and ECG study with no histology. This study was designed to solely investigate the imaging biomarker of stress perfusion, and concomitant echocardiography (unless clinically indicated) or symptom questionnaires were not taken at the time. All disease subjects (ApHCM and ASH HCM) were recruited from specialist cardiomyopathy services; therefore, any potential selection bias was consistent across the study group and disease control group. Genotyping was only performed in 47%. Eight percent of ApHCM subjects did not have long-axis perfusion imaging performed. Only vasodilator stress was performed (no exercise or inotropic stress). Coronary imaging was only performed for clinical reasons; when done, it was negative in all, and no HV had coronary assessment.

To conclude, apical perfusion defects are a ubiquitous feature of ApHCM, occurring in both mild (pre-LVH) and advanced disease (scar and aneurysms). The 100% prevalence suggests that ischemia is tightly associated with the pathophysiology of apical hypertrophy. Future work should investigate this further, as a potential phenotype accelerator, for risk stratification and as a potential therapeutic target.

## Article Information

### Sources of Funding

R.K. Hughes is supported by the British Heart Foundation (grant number FS/17/82/33222). Dr Davies is funded by the British Heart Foundation Accelerator Award (AA/18/6/34223). J.W. Malcolmson is funded by a National Institute of Health Research Clinical Doctoral Research Fellowship (ICA-CDRF-2016-02-068). Dr Lopes is funded by a Medical Research Council Clinical Academic Research Partnership award. Dr Captur is supported by the National Institute for Health Research Rare Diseases Translational Research Collaboration (NIHR RD-TRC, No. 171603) and by National Institute for Health and Care Research (NIHR) University College London Hospitals Biomedical Research Centre. This research was supported, in part, by the Intramural Research Program of the National Institutes of Health (NIH) and National Heart, Lung and Blood Institute (NHLBI) project ZIA HL006242-02. The other authors report no conflicts.

### Disclosures

None.

### Supplemental Material

Supplemental Results

Tables S1–S6

Figure S1

## Supplementary Material
