# Supplementary material for: Apical Ischemia Is a Universal Feature of Apical Hypertrophic Cardiomyopathy
Source: Circ Cardiovasc Imaging. 2023 Mar 21;16(3):e014907. doi: 10.1161/CIRCIMAGING.122.014907 (PMC10026964; doi:10.1161/CIRCIMAGING.122.014907)
Supplement: Supplementary file 1 [file hci-16-e014907-s001.pdf]

SUPPLEMENTAL MATERIAL

Supplemental Results

Medications

Cardiac medications were taken in 75% of ApHCM and 54% of ASH HCM patients. 43/100 (43%) ApHCM and 16/50 (32%) ASH HCM subjects took beta-blockers. 9/100 (9%) of ApHCM and 14/50 (28%) ASH HCM subjects took non-dihidropyridine calcium channel blockers (NDCCB). Other cardiac medications (ace-inhibitors, calcium channel blockers, diuretics) or cholesterol-lowering agents were taken in 52/100 (52%) ApHCM and 16/50 (32%) ASH HCM subjects.

Electrocardiogram analysis

Contemporaneous ECGs were available for review in 90/100 ApHCM patients. 5 had right/left bundle branch block (RBBB/LBBB) precluding T-wave assessment. TWI was present in 84/85(98.8%) which was precordial (100%), as well as lateral (89%) and inferior (31%). The one subject without TWI had V4 ST-elevation (in the context of an apical aneurysm). Maximum T-wave depth correlated with maximum R-wave amplitude ($r=0.561$, $P<0.001$). 47/50 ASH HCM patients had ECGs available for review, with 5 subjects having RBBB/LBBB precluding T-wave assessment. Precordial TWI was present in 10/42(24%), with non-specific TWI in 12/42 (single/non-contiguous leads) and no TWI in 20/42. Overall, the depth of the T-waves was significantly greater in the ApHCM cohort (8[5-10]mm vs 0[0-3]mm, $P<0.001$), as was the maximum R-wave amplitude (22[19-28]mm vs 16.5[9-21]mm, $P<0.001$).

Supplemental Table 1. Demographic and baseline CMR characteristics of overt and relative ApHCM, ASH HCM and healthy volunteers

Category	Overt ApHCM	Relative ApHCM	P value	ASH	P values<br>overt ApHCM vs ASH;<br>relative vs ASH	HV	P values<br>overt vs HV;<br>relative vs HV
**N**	68	32		50		40	
**N (%) male**	50 (73.5)	27 (84.4)	0.23	29 (58)	0.076<br>**0.012†**	23 (57.5)	0.086<br>**0.014†**
**Age at time of scan**	58.6±13	55.4 ± 14	0.29	51.8 ± 15	**0.013†**<br>**0.288†**	42.9 ± 15	**<0.001†**<br>**<0.005†**
**BSA (m^2)**	2.02±0.3	1.97 ± 0.2	0.31	2.01±0.2	0.809<br>0.375	1.94±0.2	0.112<br>0.629
**Diabetes, n (%)**	13 (19.1)	1 (3.1)	0.057	6 (12)	0.223<br>0.197	0 (0)	**<0.005†**<br>0.237
**Hypertension, n (%)**	28 (41.2)	6 (18.8)	**0.037**	14 (28)	0.072<br>0.471	0 (0)	**<0.001†**<br>**<0.005†**
**Hypercholesterolemia, n (%)**	31 (45.6)	9 (28.1)	0.118	10 (20)	**<0.005†**<br>0.269	0 (0)	**<0.001†**<br>**<0.001†**
**LA area indexed (cm^2/m^2)**	14.4 (12-17)	13.3 (12-16)	0.42	11.6 (8 – 15)	**<0.001†**<br>**0.007†**	12.3 (11 -13)	**<0.001†**<br>**<0.005†**
**LVEDVi (cm^2/m^2)**	72.2 (67–81)	74.0 (67-84)	0.52	79.1 (67 – 87)	0.078<br>0.441	76.4 (70–82)	0.081<br>0.510
**LVESVi (cm^2/m^2)**	15.6 (11–20)	17.2 (14–22)	0.083	19.1 (14 – 27)	**0.012†**<br>0.482	24.2 (21–30)	**<0.001†**<br>**<0.001†**
**LVEF (%)**	78.0±7	75.4±6	0.053	74.2 ± 9	**0.012†**<br>0.468	67.2±6	**<0.001†**<br>**<0.001†**
**RVEF (%)**	67.6 (63–72)	62.5 (58–69)	**0.018**	60.7 (52 – 66)	**<0.001†**	57.1 (42 – 48)	0.061<br>0.257

					**0.038**†		
**SV (ml)**	111.7 (101–130)	109.8 (95–133)	0.74	114.5 (93 – 133)	0.813<br>0.714	99.2 (84–115)	**0.008**†<br>0.092
**Indexed mass (g/m^2)**	89.2 (74–103)	66.4 (59–75)	**<0.001**	72.4 (57 – 91)	**<0.005**†<br>0.065	52.2 (44–58)	**<0.001**†<br>**<0.001**†
**MWT (mm)**	20.1±4	12.5±2	**<0.001**	18.5 ± 4	**0.025**†<br>**<0.001**†	8.8±2	**<0.001**†<br>**<0.001**†
**Apical cavity systolic obliteration (mm)**	25.8 (0–32)	18.6 (14–23)	0.28	8.7 (5 – 12)	**<0.005**†<br>**<0.001**†	3.5 (2–5)	**<0.001**†<br>**<0.001**†
**Presence of aneurysm (%)**	27 (39.7)	3 (9.4)	**<0.005**	1 (2)	**<0.001**†<br>0.130	0 (0)	**<0.001**†<br>**0.048**†

† P<0.05

Supplemental Table 2. Perfusion, mapping, and late gadolinium enhancement parameters of overt and relative ApHCM vs ASH HCM and healthy controls.

Category	Overt ApHCM	Relative ApHCM	P value	ASH	P value overt/relative	HV	P value overt/relative
**N**	68	32		50		32	
**Perfusion defect present**	68 (100)	32 (100)	-	45 (90)	**0.028†**<br>0.29	0 (0)	**<0.001†**<br>**<0.001†**
**Apical perfusion defect**	68 (100)	32 (100)	-	18 (36)	**<0.001†**<br>**<0.001†**	0 (0)<br>0 (0)	**<0.001†**<br>**<0.001†**
**Basal perfusion defect**	28 (41.2)	10 (31.3)	0.31	45 (90)	**<0.001†**<br>**<0.001†**	0 (0)<br>0 (0)	**<0.001†**<br>**<0.001†**
**Global stress MBF (ml/g/min)**	1.63 (1.4 – 1.9)	2.04 (1.7 – 2.2)	**<0.001†**	1.9 (1.6 – 2.3)	**<0.005†**<br>0.35	2.75 (2.3 – 3.2)	**<0.001†**<br>**<0.001†**
**Global rest MBF**	0.76 (0.6 – 0.9)	0.69 (0.6 – 0.8)	0.34	0.82 (0.6 – 1.1)	0.076<br>**0.024†**	0.87 (0.8 – 1.0)	**0.005†**<br>**<0.001†**
**Global MPR**	2.18 (1.7 – 2.6)	2.70 (2.4 – 3.5)	**<0.001†**	2.23 (1.7 – 2.9)	0.49<br>**0.006†**	3.15 (2.7 – 3.9)	**<0.001†**<br>**0.028†**
**Global subendo:subepi ratio**	0.79 (0.7 – 0.9)	0.86 (0.8 – 0.9)	**0.005†**	0.80 (0.7 – 0.9)	0.70<br>**0.016†**	0.91 (0.9 – 1.0)	**<0.001†**<br>**<0.005†**
**Apical stress MBF**	1.20 (0.9 – 1.5)	1.57 (1.4 – 1.9)	**<0.001†**	1.89 (1.5 – 2.4)	**<0.001†**<br>**0.032†**	2.73 (2.4 – 3.4)	**<0.001 †**<br>**<0.001†**
**Apical rest MBF**	0.74 (0.6 – 0.9)	0.69 (0.6 – 0.8)	0.432	0.85 (0.6 – 1.1)	**0.028†**<br>**0.014†**	0.91 (0.8 – 1.1)	**<0.001†**<br>**<0.001†**
**Apical MPR**	1.61 (1.2 – 2.0)	2.27 (1.8 – 3.0)	**<0.001†**	2.30 (1.7 – 2.9)	**<0.001†**<br>0.926	3.08 (2.6 – 3.7)	**<0.001†**<br>**<0.001†**
**Apical subendocardial stress MBF**	0.82 (0.7 – 1.1)	1.31 (1.0 – 1.8)	**<0.001†**	1.69 (1.2 – 2.2)	**<0.001†**<br>**0.049†**	2.51 (2.2 – 3.0)	**<0.001†**<br>**<0.001†**

**Apical subendocardial rest MBF**	0.69 (0.6 – 0.9)	0.68 (0.6 – 0.8)	0.62	0.83 (0.7 – 1.0)	**0.022**†<br>**0.031**†	0.90 (0.7 – 1.1)	**<0.001** †<br>**<0.005**†
**Apical subendo:subepi ratio**	0.64 (0.5 – 0.8)	0.77 (0.7 – 0.9)	**<0.001**†	0.84 (0.7 – 0.9)	**<0.001**†<br>0.614	0.91 (0.8 – 1.1)	**<0.001** †<br>**<0.001**†
**Apical subendocardial stress flow lower than rest n(%)**	25 (36.7)	3 (9.4)	**<0.005**†	4 (8.9)	**<0.001**†<br>0.87	0 (0)	**<0.001**†<br>**0.048**†
**Apical:basal stress MBF ratio**	0.66 (0.5 – 0.8)	0.75 (0.6 – 0.9)	**0.009**†	1.08 (0.9 – 1.4)	**<0.001**†<br>**<0.001**†	1.14 (1.0 – 1.3)	**<0.001**†<br>**<0.001**†
**Subendo apical: basal stress MBF ratio**	0.57 (0.4 – 0.8)	0.71 (0.5 – 0.9)	**0.017**†	1.13 (0.9 – 1.6)	**<0.001** †<br>**<0.001**†	1.00 (1.0 – 1.1)	**<0.001**†<br>**<0.001**†
**Global T1**	1057.1 (1025 – 1073)	1010.4 (991 – 1036)	**<0.001**†	1023.5 (1002 – 1049)	**<0.005**†<br>0.083	999.7 (982 – 1020)	**<0.001**†<br>**0.045**†
**Global T2**	49.0±2	46.8±2	**<0.001**†	48.3±2	0.23<br>**0.036**†	48.2±2	**0.024**†<br>**0.031**†
**Global ECV**	26.6±3	25.0±3	**0.028**†	26.0±3	0.34<br>**0.039**†	24.9±2	**0.015**†<br>0.93
**LGE present n (%)**	60 (88.2)	12 (37.5)	**<0.001**†	21 (42)	**<0.001**†<br>0.818	0 (0)	**<0.001**†<br>**<0.001**†
**LGE g FWHM**	23.8 (14 – 37)	0.0 (0 – 7)	**<0.001**†	0.0 (0 – 14)	**<0.001**†<br>0.42	0.0 (0 – 0)	**<0.001**†<br>**<0.001**†
**LGE % FWHM**	17.4 (11 – 24)	0.0 (0 – 5)	**<0.001**†	0.0 (0 – 15)	**<0.001**†<br>0.57	0.0 (0 – 0)	**<0.001**†<br>**<0.001**†
**Apical LGE relative enhanced area (%) FWHM**	30.0 (19 - 41)	0.0 (0 - 20)	**<0.001**†			0.0 (0 - 0)	**<0.001**†<br>**<0.001**†

ApHCM = apical hypertrophic cardiomyopathy, ASH = asymmetrical septal hypertrophy, HV = healthy volunteer, MBF = myocardial blood flow, MPR = myocardial perfusion reserve, ECV = extracellular volume, LGE = late gadolinium enhancement

† P <0.05

Supplemental Table 3. Comparison of CMR parameters between pure and mixed phenotypes of apical hypertrophic cardiomyopathy

	Pure ApHCM	Mixed ApHCM	P value
**N**	39	29	
**N (%) male**	28 (71.8)	22 (75.9)	0.707
**Age at time of scan**	61.0±13.7	55.4±12.4	0.084
**BSA (m2)**	2.04±0.3	1.99±0.3	0.444
**LA area indexed (cm^2/m^2)**	13.8 (12-17)	14.8 (13-17)	0.539
**LVEDVi (cm^2/m^2)**	71.4 (66-78)	73.1 (68-85)	0.343
**LVESVi (cm^2/m^2)**	16.2 (12-21)	15.3 (11-18)	0.224
**LVEF (%)**	76.6±1	79.8±6	**0.043**†
**RVEF (%)**	68.6 (65-72)	66.5 (62-72)	0.400
**SV (ml)**	110.5 (102-121)	115.2 (100-144)	0.292
**Indexed mass (g/m^2)**	79.3 (72-94)	100.7 (84-122)	**<0.001**†
**MWT (mm)**	19.7±4.1	20.7±3.2	0.248
**Apical cavity systolic obliteration (mm)**	25.8 (0-30.8)	25.9 (7-32)	0.656
**Presence of aneurysm (%)**	16 (41)	11 (38)	0.796
**Apical perfusion Defect, n (%)**	39 (100)	29 (100)	-
**Basal perfusion Defect, n (%)**	14 (36.8)	14 (48.3)	0.347
**Global stress MBF (ml/g/min)**	1.65 (1.4-1.9)	1.62 (1.4-1.8)	0.611
**Global rest MBF**	0.76 (0.6-0.9)	0.74 (0.6-0.9)	0.573
**Global MPR**	2.09 (1.7-2.8)	2.23 (1.9-2.5)	0.828
**Global subendo:subepi ratio**	0.81 (0.7-0.9)	0.76 (0.7-0.9)	0.491
**Apical stress MBF**	1.15 (0.9-1.5)	1.23 (1.0-1.5)	0.446
**Apical rest MBF**	0.79 (0.6-0.9)	0.71 (0.6-0.9)	0.710
**Apical MPR**	1.48 (1.2-1.9)	1.66 (1.2-2.1)	0.556
**Apical subendocardial stress flow lower than rest n(%)**	13 (33.3)	12 (42.9)	0.427

**Apical:basal stress MBF ratio**	0.60 (0.5-0.8)	0.73 (0.6-0.8)	**0.032†**
**Subendo apical: basal stress MBF ratio**	0.64 (0.6-0.8)	0.63 (0.5-0.8)	0.382
**Global T1**	1044.6 (1015-1067)	1063.5 (1043-1080)	**0.021†**
**Global T2**	48.8±2.6	49.2±2.1	0.490
**Global ECV**	26.7±3.6	26.5±3.2	0.802
**LGE present n (%)**	35 (89.7)	25 (86.2)	0.715
**LGE g FWHM**	20.8 (12-32)	26.7 (10-38)	0.394
**LGE % FWHM**	17.3 (9-25)	15.4 (7-21)	0.208
**Apical LGE relative enhanced area (%) FWHM**	33.0 (21-44)	24.1 (16-32)	**0.041†**

ApHCM = apical hypertrophic cardiomyopathy, BSA = body surface area, ECV = extracellular volume, FWHM = full width half maximum, LA = left atrium, LGE = late gadolinium enhancement, LVEDVi = indexed left ventricular end-diastolic volume, LVESVi = indexed left ventricular end-systolic volume, LVEF = left ventricular ejection fraction, MBF = myocardial blood flow, MPR = myocardial perfusion reserve, MWT = maximum wall thickness, RVEF = right ventricular ejection fraction, SV = stroke volume

†P <0.05

Supplemental Table 4. Description of distribution of apical late gadolinium enhancement in different apical hypertrophic cardiomyopathy morphological subtypes

Distribution N (%)	Relative ApHCM N=32	Mixed ApHCM N=29	Pure ApHCM N=39	ApHCM micro-aneurysm N=16	ApHCM aneurysm N=14
**Nil**	20 (63)	4 (14)	4 (10)	0 (0)	0 (0)
**Subendocardial/patchy**	8 (25)	4 (14)	9 (23)	1 (6)	0 (0)
**Subendocardial/dense**	0 (0)	6 (21)	4 (10)	3 (19)	2 (14)
**Transmural/patchy**	2 (6)	5 (17)	8 (21)	2 (12)	2 (14)
**Transmural/dense**	2 (6)	10 (34)	14 (36)	10 (63)	10 (72)

ApHCM = apical hypertrophic cardiomyopathy

Supplemental Table 5. Univariate associations of clinical and demographic variables with apical stress myocardial blood flow (MBF) in patients with overt and relative ApHCM

	**Regression (beta) coefficient**	**95% CI**	**P-value**
**Age**	-0.005	-0.01, 0.00	0.123
**Sex**	0.143	-0.075, 0.361	0.196
**BSA (m^2)**	0.022	-0.38, 0.42	0.915
**LA area indexed (cm^2/m^2)**	0.013	-0.02, 0.04	0.358
**Apical cavity obliteration (mm)**	0.001	-0.01, 0.01	0.727
**MWT (mm)**	-0.050	-0.07, -0.03	**<0.001†**
**Indexed LV mass (g/m^2)**	-0.005	-0.01, -0.00	**0.010†**
**Presence of apical aneurysm**	-0.192	-0.39, 0.01	0.057
**LVEDVi (cm^2/m^2)**	0.007	0.00, 0.01	**0.039†**
**LVESVi (cm^2/m^2)**	0.024	0.01, 0.04	**<0.001†**
**LVEF (%)**	-0.027	-0.04, -0.01	**<0.001†**
**RVEF (%)**	-0.015	-0.03, -0.00	**0.012†**
**Apical rest MBF (ml/g/min)**	0.087	-0.49, 0.66	0.763
**Global T1 (ms)**	-0.005	-0.01, -0.00	**<0.001†**
**Global T2 (ms)**	-0.054	-0.10, -0.01	**0.011†**
**Global ECV (ms)**	-0.022	-0.05, 0.01	0.176
**Apical LGE (%)**	-0.007	-0.01, -0.02	**0.006†**
**T-wave depth (mm)**	-0.036	-0.07, -0.01	**0.017†**
**R wave amplitude (mm)**	-0.023	-0.04, -0.01	**0.001†**

BSA = body surface area, ECV = extracellular volume, LA = left atrium, LGE = late gadolinium enhancement, LV = left ventricular, LVEDVi = indexed left ventricular end diastolic volume, , LVEF = left ventricular ejection fraction, LVESVi = indexed left ventricular end systolic volume, MBF = myocardial blood flow, MWT = maximum wall thickness, RVEF = right ventricular ejection fraction.

†$P<0.05$

Supplementary Table 6. Multivariable linear regression model for clinical and demographic variables associated with apical stress myocardial blood flow (MBF) in patients with overt and relative ApHCM.

Independent variable	Beta (correlation) coefficient	95% confidence interval	VIF	P value
**Age**	-0.011	-0.02, -0.00	1.476	**0.014**†
**Sex**	-0.060	-0.31, 0.19	1.453	0.627
**BSA (m^2)**	-0.359	-0.87, 0.15	1.433	0.162
**MWT (mm)**	-0.031	-0.06, -0.01	1.961	**0.013**†
**LVEF (%)**	-0.025	-0.04, -0.01	1.222	**<0.005**†
**RVEF (%)**	-0.010	-0.02, 0.00	1.147	0.075
**T1 (ms)**	-0.001	-0.00, 0.00	2.140	0.717
**T2 (ms)**	0.004	-0.04, 0.05	1.677	0.871
**Apical LGE (%)**	-0.004	-0.01, 0.0	1.526	0.107
**R wave amplitude (mm)**	-0.023	-0.04, -0.01	1.351	**<0.005**†

BSA = body surface area, LGE = late gadolinium enhancement, LVEF = left ventricular ejection fraction, MWT = maximum wall thickness, RVEF = right ventricular ejection fraction.

†$P<0.05$

Supplemental Figure 1. Distribution of apical late gadolinium enhancement. The 2-chamber cine in end-diastole is provided for reference. No LGE is present in **A** -shown in the apical short axis (**i**) and 2-chamber (**ii**). **B** demonstrates subendocardial/patchy LGE. **C** demonstrates subendocardial/dense LGE. **D** demonstrates transmural/patchy LGE and **E** demonstrates transmural/dense LGE. Corresponding stress perfusion maps shown (**iv**).

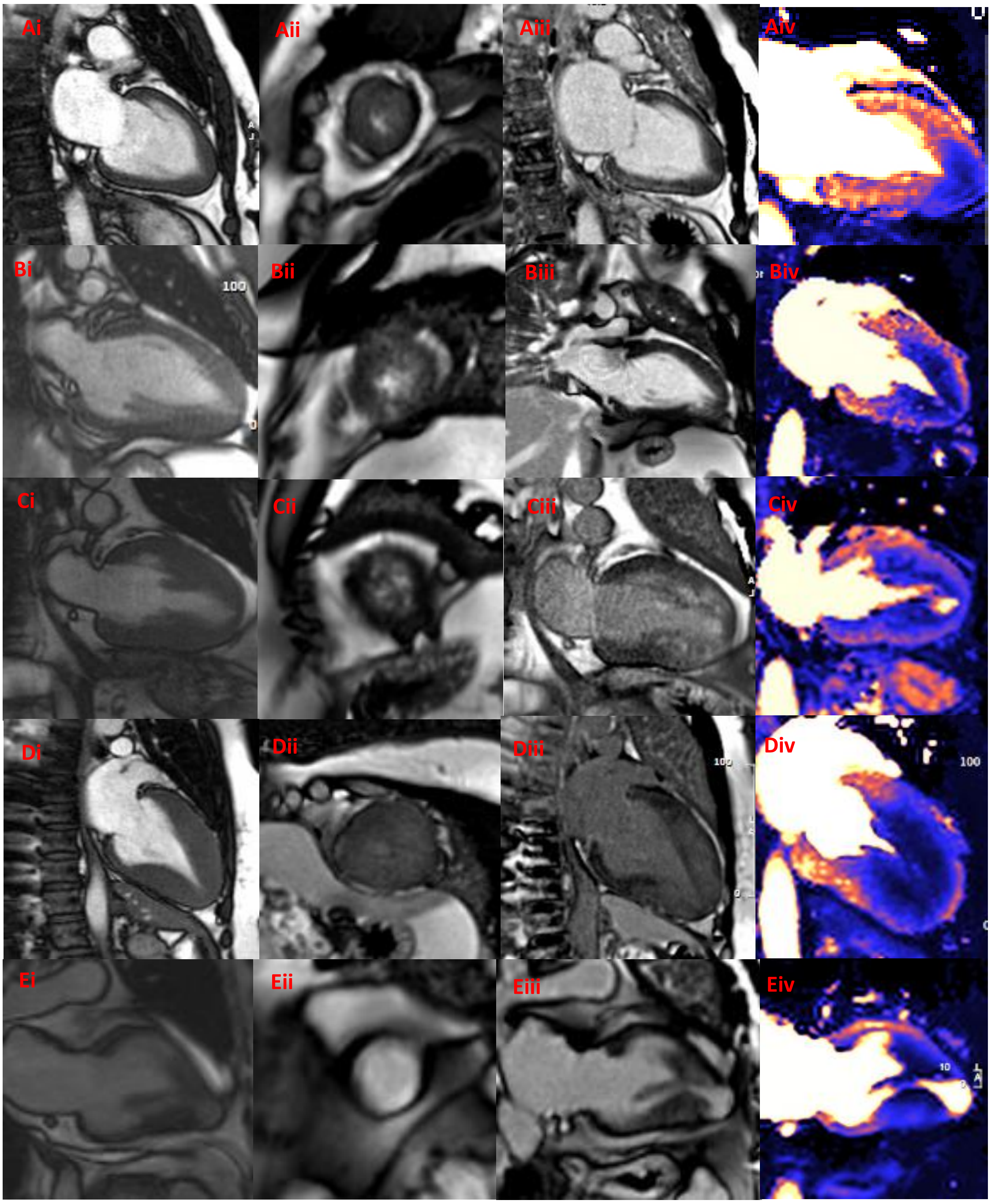

LGE = late gadolinium enhancement
